# Postictal resting-state connectivity changes after electroconvulsive therapy-induced seizures

**DOI:** 10.1007/s00406-025-02043-7

**Published:** 2025-07-14

**Authors:** Julia C. M. Pottkämper, Joey P. A. J. Verdijk, Sven Stuiver, Freek ten Doesschate, Michel J. A. M. van Putten, Jeannette Hofmeijer, Jeroen A. van Waarde, Guido A. van Wingen

**Affiliations:** 1 Clinical Neurophysiology Group, University of Twente, Enschede, The Netherlands; 2 Department of Psychiatry, Rijnstate Hospital, Arnhem, The Netherlands; 3 Department of Neurology, Rijnstate Hospital, Arnhem, The Netherlands; 4 Department of Psychiatry, Amsterdam UMC Location University of Amsterdam, Amsterdam, The Netherlands; 5 Department of Neurology and Clinical Neurophysiology, Medisch Spectrum Twente, Enschede, The Netherlands

**Keywords:** Functional magnetic resonance imaging, Postictal state, Depression, Electroconvulsive therapy, Healthy controls, Resting state networks

## Abstract

Electroconvulsive therapy (ECT) leads to temporary changes of brain function. It is unclear what changes take place shortly after the induced seizures. Here, we present the first human study on resting-state network (RSN) changes in the immediate postictal state. The objective was to investigate brain-wide RSNs connectivity changes shortly after ECT-induced seizures. We analyzed prospectively collected resting-state functional magnetic resonance imaging scans from 17 patients with major depression at baseline and one hour after ECT-sessions. RSNs were extracted and changes in mean and voxel-wise RSN connectivity strength were calculated. Data were compared to 27 age, sex, and level of education matched healthy individuals to account for test–retest effects. Clinical postictal recovery was measured using the reorientation time questionnaire. Group by time interaction analyses showed postictal decreases in mean connectivity strength in the left central executive network and the auditory network in patients, compared to retest in healthy controls (β = − 0.18 [CrI95 − 0.27, − 0.09] and (β = − 0.22 [CrI95 − 0.36, − 0.07], respectively). Voxel-wise analyses revealed increased between-network connectivity in the salience network with cerebellar regions compared to healthy controls, along with decreased within-network connectivity in the default mode network and left central executive network. No significant associations with clinical recovery or other variables were observed. In this cohort, ECT-induced seizures were followed by postictal decreases of connectivity strength in the left central executive network and the auditory network and increase of connectivity between the salience network and cerebellum. Postictal network changes were not associated with clinical postictal recovery.

*Clinical trial registration:* ClinicalTrials.gov NCT04028596.

**Supplementary Information:**

The online version contains supplementary material available at 10.1007/s00406-025-02043-7.

## Introduction

Electroconvulsive therapy (ECT) is an effective antidepressive treatment for patients with depression, in which generalized seizures are induced. It often results in improved mood after several sessions [1, 2]. Immediately after the ECT-session, patients may experience side-effects such as impaired consciousness, fatigue, headaches, nausea, muscle aches, motor restlessness, agitation or delirium, that presumably are the consequence of the electrically induction, propagation and termination of the seizure activity [1, 3, 4]. Occurrence and severity of side-effects are determined by manipulation of electrode placement, electrical charge, stimulus pulse width, current amplitude and treatment frequency [5]. Despite the relevance of seizures for the antidepressant action of ECT, there is limited knowledge about how seizures influence brain function during the postictal state, which could provide new leads for the mitigation of cognitive side-effects.

The postictal state may affect functional brain connectivity, which can be investigated with resting-state functional magnetic resonance imaging (rs-fMRI). With rs-fMRI, so-called resting state networks (RSNs) can be extracted with independent component analysis (ICA), that are anatomically separate but functionally connected brain regions [6, 7]. RSNs serve as a proxy for synchronous neuronal activity intrinsically generated by the brain at rest (i.e., without a specific task), which allows to investigate the brain’s functional architecture [6, 8]. Also, RSNs are associated with human consciousness, attention, memory, perception, mood, (introspective) thinking, learning, decision making, motor functioning and language [7], that are all cognitive and motor processes that are affected during the postictal state.

Neuroimaging studies of the immediate postictal state after an ECT-induced seizure are non-existent. Instead, rs-fMRI have focused on investigating brain connectivity after a successful ECT-course, when postictal symptoms have subsided. For example, we and others showed that within- and between-network connectivity involving the default mode network (DMN) increased compared to changes in healthy controls [9, 10]. Increased connectivity of the left central executive network (CEN) was associated with higher treatment effectiveness [9]. Because preclinical studies have shown that induced seizures cause vasoconstriction-mediated hypoperfusion [11], we also studied postictal cerebral blood flow (CBF) in ECT-patients. In the immediate postictal state (i.e., one hour after the seizure), we showed lower postictal CBF when ECT-induced seizures appeared longer in duration [12]. On the other hand, after short seizures, the postictal CBF actually increased [12]. Even though there are clearly visible effects on perfusion, it remains uncertain how this affects functional brain connectivity in the early postictal state and how this may relate to clinical symptoms.

To date, only one animal study investigated rs-fMRI in rats within the first minutes postictally. This study found widespread postictal cortical blood oxygenation level dependent (BOLD) decreases, mostly in the hippocampus [13]. It is unknown if these BOLD decreases translate to the human postictal state or if changes in functional connectivity can be detected. We aimed to study changes in RSNs directly after seizures in humans. To achieve this, we acquired rs-fMRI scans at approximately one hour after ECT-induced seizures and investigated postictal changes in twelve large-scale canonical RSNs compared to baseline, controlled for test–retest effects in healthy controls. Functional connectivity changes and their relationship with clinical postictal recovery were investigated.

## Methods

### Study design

This is a post hoc analysis with rs-fMRI data of a prospective clinical trial with three-condition randomized cross-over design (i.e., the SYNAPSE-trial, in which patients participated in two medications and one placebo condition; approved by the local medical-ethical authority with no. NCT04028596; protocol and primary outcomes are described elsewhere) [14]. For the current analyses, we used rs-fMRI scans at baseline (i.e., < 1 week before the ECT-course) and ~ 1 h after ECT-induced seizures in the placebo condition (i.e., 50 cc water), as measure of the immediate postictal state. To interpret our results in patients, we controlled for test–retest effects using two separate rs-fMRI measures from healthy controls (i.e., baseline and 1 month follow-up), scanned with the same MRI hardware and scanning protocols.

### Participants

Patients were included if aged ≥ 18 years, classified having major depressive episode according to the Mini International Neuropsychiatric Interview (MINI) [15] and treated with ECT at Rijnstate Hospital, Arnhem, The Netherlands. Exclusion criteria were chronic use of acetaminophen, calcium antagonists, or non-steroid anti-inflammatory drugs, and contraindications for undergoing MRI [14]. Healthy controls had no history of psychopathology according to the MINI and were matched to our patients regarding age, sex, and level of education. All participants were Dutch speaking and gave oral and written informed consent.

Clinical recovery after seizures was assessed with the reorientation time questionnaire (ROT) comprising five items [16]. Patients were asked to reproduce their name, age, birthday, current location (i.e., the hospital’s name), and the day of the week. A score in minutes was assigned based on the number of correct responses out of five questions, with a minimum threshold of four correct answers compared to baseline responses. The resulting scores varied between 5 and 100 min.

Details of the EEG procedure can be found in the supplementary.

### ECT procedure

ECT was administered according to the Dutch treatment guideline, using a Thymatron System IV device (Somatics Incorporation Lake Bluff, Illinois, USA), delivering a stimulus with constant-current (0.9 Ampѐre) in bidirectional, square waves and in brief pulses (1 ms). Electrode placement included unilateral (UL; according to d’Elia [17]) or bifrontotemporal (BL; also known as bitemporal). Intravenously, patients received anesthesia (etomidate 0.2–0.3 mg/kg) and proper muscle relaxation (succinylcholine 0.5–1 mg/kg), and were pre-oxygenated (100% O_2_, positive pressure) until resumption of spontaneous respiration. In case of severe postictal confusion or motor restlessness, 2.5–5 mg midazolam was administered intravenously. Pre- and post-ECT medication was kept constant in the context of current care and left to the discretion of the treating psychiatrist (e.g., antidepressant, antipsychotic, analgesic) [18].

### Imaging data acquisition, preprocessing, and analyses

#### Data acquisition

High-resolution T_1_-weighted (T_1_W) and rs-fMRI data were acquired at baseline and in the postictal state (or in healthy controls after one month), using a 3 T Philips Achieva scanner (Philips Healthcare, Best, The Netherlands) equipped with a SENSE eight-channel receiver head coil. The scanning protocol included a high-resolution T_1_W turbo field echo MRI (sequence parameters = TR 7.5 ms, TE 4.6 ms, flip angle 8°, 145 sagittal slices, voxel size 1.1 mm isotropic, scan duration 5.5 min) and rs-fMRI (sequence parameters = TR 1981 ms, TE 27 ms, flip angle 90°, voxel size 3.0 × 3.0 × 3.0 mm, FOV 240 mm, 160 volumes scanned in two packages, total scan duration 10 min). During the rs-fMRI scans, patients and healthy controls were instructed to relax and stay awake.

#### Preprocessing

Preprocessing was performed using a singularity image container running *fMRIPrep* (v21.0.2, https://fmriprep.org/en/21.0.2/) [19]. *fMRIPrep* uses a standardized procedure involving generation of a reference volume, co-registration to the T_1_W image, motion correction (ICA-AROMA), and normalization to MNI space (MNI152NLin2009cAsym) using a combination of all spatial transformations. *fMRIPrep’s* non-aggressive denoised motion corrected output was verified by visual inspection of the individual reports. Our subsequent preprocessing pipeline consisted of (i) skull stripping, (ii) spatial smoothing with an isotropic, Gaussian kernel of 6 mm full-width-at-half-maximum, (iii) nuisance regression (regressing out average white matter and cerebrospinal fluid signals to exclude physiological noise), (iv) removal of first 5 non-steady state volumes, and (v) high-pass filtering by 0.007 Hz. Volumes with excessive movement (i.e., framewise displacement of > 3 mm were removed from BOLD timeseries). Detailed information about the *fMRIPrep* pipeline is presented in the Supplementary material. After preprocessing, quality control was performed, selecting scans that retained at least 4 min of sufficiently motion-free data, with a mean framewise displacement of < 0.6 mm.

#### Spatially constrained independent component analysis

A multivariate-objective optimization ICA with reference (MOO-ICAR) algorithm was used [20, 21]. This analysis has been conducted within the Group ICA for fMRI toolbox (icatb.sourceforge.net) [20]. In MOO-ICAR, first, twelve large-scale brain networks from another study including 160 healthy controls were used as templates to extract independent components (ICs) on the subject and session level [21–23]. This analysis preserved independence of ICs at the subject level while ensuring correspondence of ICs across subjects and enables more accurate longitudinal ICA analyses [21]. Twelve large-scale resting state networks were selected for our further analysis, with the DMN, attention network (ATN), salience network (SN), and CEN as primary networks of interest based on their involvement in depressive disorders and possible changes after ECT [9, 22, 24–26]. We expected decreased connectivity in these networks, based on the assumption that the postictal state leads to disruptions in cerebral blood flow [12].

#### Mean network connectivity strength

Mean network connectivity strength within each RSN was investigated with average Z-scores, reflecting the magnitude of functional connectivity within a RSN [27]. We binarized all group-level RSNs (Z-score > 1), which were combined with the subject specific RSNs. The mean of all voxels within an RSN was calculated, yielding one Z-score per participant and per time point (i.e., baseline, postictal, follow-up) for each RSN. Difference RSN maps between two time points were calculated and then overlayed with the binarized group-level RSNs.

### Statistical analyses

For all clinical, demographic, and mean network connectivity strength data, the statistical program R version 4.2.3 was used [28]. Quantitative variables were reported as medians with interquartile ranges (IQR). Patients and healthy controls were compared with respect to age, sex, and level of education with *t* tests and chi-square tests, where appropriate. *P* values < 0.05 were considered statistically significant.

We investigated mean network strength with Bayesian regression models to explore group by time interaction effects (i.e., patients and healthy controls corrected for age and sex) and relations with clinical variables (i.e., seizure duration, electrode placement, and time interval between ECT-stimulus and rs-fMRI acquisition) in the RSNs of interest (DMN, ATN, SN, CEN), and exploratively in the remaining RSNs, using the package brms [29]. Exploratory regression analyses were used to investigate the effect of the use of postictal midazolam on mean network strength per resting state network. In the Bayesian analyses, we used 4 chains with 2000 draws of the posterior distribution per chain. A Gaussian likelihood and default priors were used for the beta coefficients. The first 1000 draws of each chain were considered warmup and therefore discarded. Beta coefficients that were larger or smaller than 0 with a probability of 95% were considered credible (which may compare with the label ‘significant’ in frequentist statistics). For equivalence testing, we used a region of practical equivalence (ROPE) of (− 0.02, 0.02) based on the standard deviation of the outcome variables. If 95% of the posterior beta coefficient fell within the ROPE, equivalence was inferred [30]. Effect sizes will be displayed as Bayes R^2^ and will be interpreted according to Cohen with 0.13–0.26 interpreted as medium effect and ≧ 0.26 as large effect [31].

**Fig. 1:**
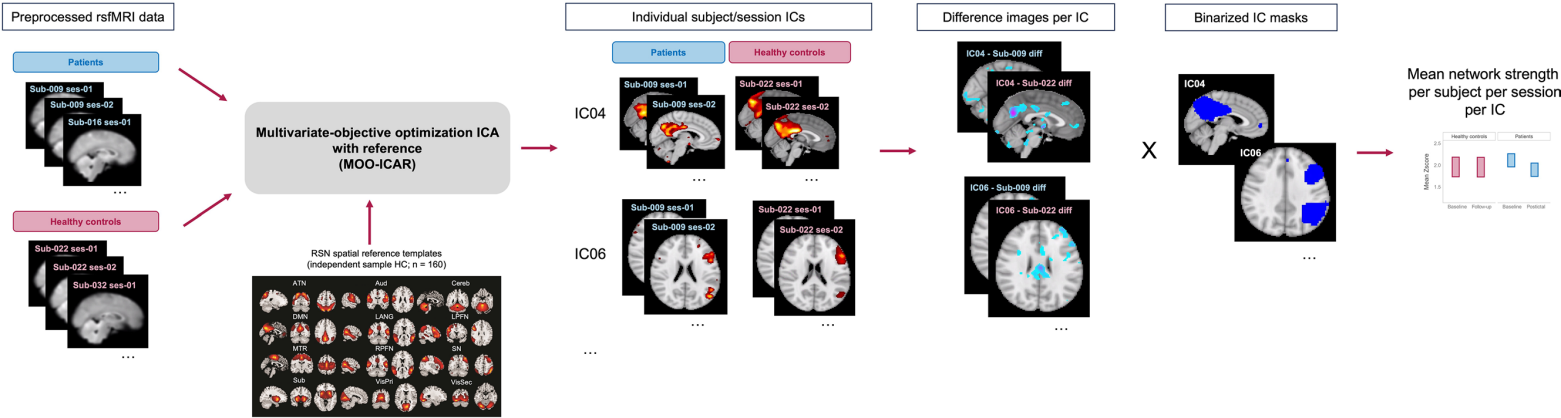
Flowchart depicting resting-state functional magnetic resonance imaging (rs-fMRI) data analyzed with the multivariate-objective optimization independent component (IC) analysis with reference (MOO-ICAR; A) algorithm, yielding 12 canonical large-scale resting state networks (RSNs; B). Mean network strength was computed per resting state network (i.e., IC), using individual subject ICs overlayed with binarized IC masks (*Z*-score > 1) of each IC. A detailed overview of all RSNs can be found in the supplementary material (Fig. S1). *ATN* attention network, *Aud* auditory network, *Cereb* cerebellar network, *DMN* default mode network, *LANG* language network, *LCEN* left central executive network, *MTR* somatosensory network, *RCEN* right central executive network, *SN* salience network, *Sub* subcortical network, *VisPri* primary visual network, *VisSec* secondary visual network

To investigate voxel-wise changes between baseline and postictal RSNs, controlled for test–retest effects in healthy controls, subject specific difference RSN maps were used as input for nonparametric permutation tests in Parametric Analyses of Linear Models (PALM; www.fmrib.ox.ac.uk/fsl). First, we investigated changes in the DMN, ATN, SN, and CEN (i.e., divided in left and right CEN) between baseline and the postictal state, compared to changes in healthy controls (i.e., group by time interaction effect), with an omnibus *F*-test. In case of a significant *F*-test, one-tailed two-sample post hoc* t* tests were performed to assess simple effects. Age and sex were entered as covariates of no interest. Threshold-free cluster enhancement (TFCE) for family wise error (FWE) correction with an α-level of 0.05 and correction for multiple components (i.e., five RSNs of interest) and contrasts were applied [32]. Second, to investigate changes in RSNs in relation with seizure duration, we used separate regression models for the networks of interest, and, again, controlled for multiple components (Fig. 1). All analyses were also performed for the remaining RSNs (i.e., auditory, cerebellar, language, somatomotor, subcortical, primary and secondary visual networks) with statistical significance corrected for multiple comparisons across voxels and RSNs (*p* < 0.05, with TFCE and FWE-corrected). For all voxel-wise analyses MATLAB version 2022a was used (Natick, Ma, The Math Works, 2022 Inc.).

## Results

### Participants

Seventeen patients (median age 58 years [22 IQR], 9 females [53%]), and 27 healthy controls (median age 55 years [23 IQR], 15 females [56%]) were included, who did not differ in age (*p* = 0.225), sex (*p* = 0.691), or level of education (*p* = 0.259). Eleven patients were treated with BL electrode placement and six with UL (i.e., one with left and eight with right UL placement). At the ECT-session before the rs-fMRI acquisition, median seizure duration was 49 s (13 IQR), elicited by applying a median delivered electrical charge of 303.8 mC (251.5 IQR). After quality control, a total of 44 baseline, 17 postictal and 27 follow-up healthy control MRI scans were available for analyses. Four out of 17 patients (24%) had a maximum ROT score of 100 min, which means these patients were not reoriented at the time they had the postictal MRI scan. The other 13 patients (76%) had a median ROT of 35 min (15 IQR), which means they were fully oriented at the time of scanning. Patient and ECT characteristics are provided in Table 1.

**Table 1:** Patient and ECT characteristics

Characteristic	Patients (*N* = 17)
Age in years, median (range; IQR)	58 (21–82; 22)
Female, n (%)	9 (53)
Bifrontotemporal electrode placement^a^, n (%)	11 (65)
Median delivered charge at the ECT-session before rs-fMRI acquisition, in milliCoulombs (range; IQR)	303.8 (125.6–659.7; 251.5)
Median seizure duration at the ECT-session before rs-fMRI acquisition, in seconds (range; IQR)	49 (25–79; 13)
Median ROT at the ECT-session before rs-fMRI acquisition, in min (range; IQR)	40 (20–100; 35)
Number of patients who received postictal midazolam before rs-fMRI acquisition, n (%)	7 (41)
Median interval between the ECT-stimulus and postictal rs-fMRI image acquisition, in minutes (range; IQR)	78 (68–101; 11)

*IQR* interquartile range, *ECT* electroconvulsive therapy, *rs-fMRI* resting-state functional magnetic resonance imaging, *ROT* reorientation time

^a^Four patients were initially treated with right unilateral electrode placement, which was changed to bifrontotemporal electrode placement until the end of their ECT-course

### Mean network connectivity strength

#### Networks of interest (i.e., DMN, ATN, SN, CEN)

**Fig. 2:**
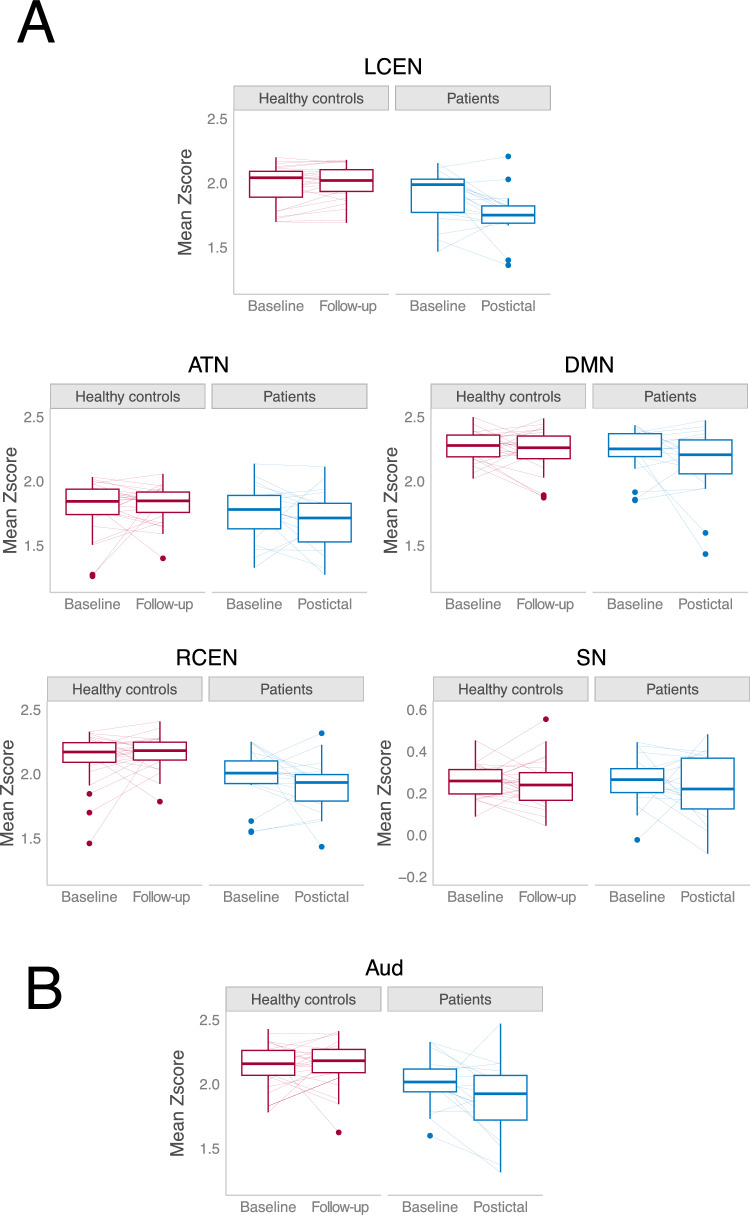
Changes in postictal mean network connectivity strength in the resting state networks (RSNs) of interest (**A**) and changes in the exploratory auditory network (**B**). Postictal mean network connectivity strength was decreased in the left central executive and auditory network in electroconvulsive therapy patients (n = 17) compared to changes over time in healthy controls (n = 27). Note that y-axis ranges for the salience network were adjusted for improved readability. *ATN* attention network, *DMN* default mode network, *LCEN* left central executive network, *RCEN* right central executive network, *SN* salience network

In the Bayesian regression models, we established a credible group by time interaction in the left CEN (β = − 0.18 [CrI95 − 0.27, − 0.09], see Table 2 and Fig. 2A), meaning that patients had lower mean network connectivity strength in the postictal state compared to baseline, relative to changes over time in healthy controls. The effect size was large, with a Bayesian R^2^ of 0.31 (CrI95 0.13, 0.47). These results were confirmed when examining mean network connectivity changes in patients (R^2^ = 0.34 [CrI95 0.15, 0.50)], controlling for clinical variables (i.e., seizure duration, electrode placement, or time interval between ECT-stimulus and rs-fMRI acquisition; see Supplementary Table S1). More specific, clinical postictal characteristics (i.e., ROT and use of midazolam) showed no credible interactions with mean network connectivity changes. None of the other networks of interest showed credible interactions (see Supplementary Table S2). Postictal midazolam did not affect change in mean network connectivity strength in any RSN (see Supplementary Table S3). In patients, none of the clinical variables were credibly related to changes in mean network connectivity strength in the remaining RSNs (see Supplementary Table S4). Between the two measurements, healthy controls showed no change of mean network connectivity strength in any of the RSNs (see Supplementary Table S5).

**Table 2:** Bayesian regression results highlighting credible group by time interactions in the left central executive and auditory networks

Predictors	Networks of interest										Other networks	
	ATN		DMN		LCEN		RCEN		SN		AUD	
	Estimates	CrI (95%)	Estimates	CrI (95%)	Estimates	CrI (95%)	Estimates	CrI (95%)	Estimates	CrI (95%)	Estimates	CrI (95%)
Intercept	− 0.13	− 0.43 to 0.17	0.04	− 0.25 to 0.32	− 0.07	− 0.25 to 0.12	0.09	− 0.20 to 0.40	0.07	− 0.12 to 0.26	− 0.17	− 0.46 to 0.12
Group (patients)	− 0.08	− 0.22 to 0.07	− 0.05	− 0.18 to 0.10	− 0.18	− 0.27 to − 0.09*	− 0.12	− 0.27 to 0.02	− 0.01	− 0.11 to 0.08	− 0.22	− 0.36 to − 0.07*
Age (years)	0.00	− 0.00 to 0.01	− 0.00	− 0.01 to 0.00	0.00	− 0.00 to 0.00	− 0.00	− 0.01 to 0.00	− 0.00	− 0.00 to 0.00	0.00	− 0.00 to 0.01
Sex (female)	0.11	− 0.04 to 0.25	− 0.05	− 0.18 to 0.09	− 0.02	− 0.11 to 0.07	0.08	− 0.06 to 0.23	0.02	− 0.08 to 0.11	0.02	− 0.12 to 0.16
R^2^					0.31	0.13 to 0.47					0.21	0.06 to 0.37
ROPE Interpretation			Credible				Credible					

*ATN* attention network, *AUD* auditory network, *DMN* default mode network, *LCEN* left central executive network, *RCEN* right central executive network, *SN* salience network, *ROPE* region of practical equivalence, *CrI* credibility interval

#### Other networks

In exploratory analyses, we established a credible postictal decrease in mean connectivity strength in the auditory network (β = − 0.22 [CrI95 − 0.36, − 0.07]), compared to healthy controls (Fig. 2B), with a medium effect size (R^2^ = 0.21 [CrI95 0.06, 0.37]). Post hoc tests in patients revealed credible decreases in mean network connectivity strength in the auditory network showing a large effect size (β = − 0.16 [CrI95 − 0.29, − 0.04]; R^2^ 0.44 [CrI95 0.25–0.58]; see Supplementary Table S1). None of the remaining RSNs showed any credible group by time interaction effects.

### Voxel-wise analyses

#### Networks of interest

**Fig. 3:**
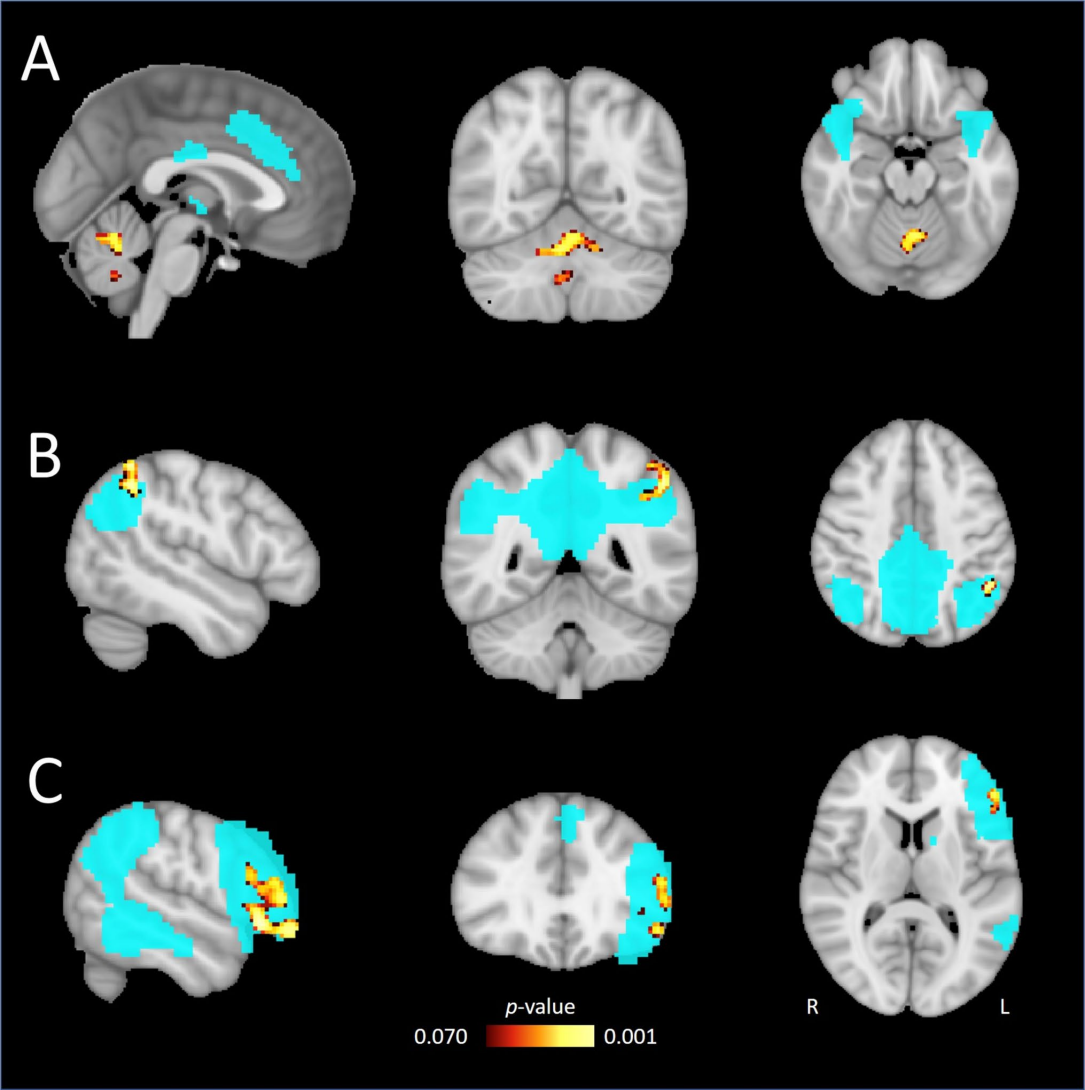
*P*-value maps of postictal voxel-wise changes in the salience network (SN; **A**), default mode network (DMN; **B**), and left central executive network (LCEN; **C**) in electroconvulsive therapy patients (n = 17) controlled for test–retest variability in healthy controls (n = 27). The blue areas represent the network masks (SN, DMN, LCEN, respectively). Post hoc comparisons of changes over time in patients showed significant effects only in the salience network, revealing increased connectivity of three clusters in the cerebellum with the salience network in the postictal state. *P*-values are depicted ranging from 0.070 to 0.001 for illustration purposes. MNI voxel location is [− 1, − 58, − 18], [− 48, − 48, 3], and [− 52, 31, 9], respectively. *R* right, *L* left

We established three significant group by time interaction effects in the SN, DMN, LCEN. We found three significant clusters in the SN (i.e., left cerebellar structures; see Table 3 and Fig. 3). Post-hoc comparisons of patients revealed an increase in postictal connectivity with cerebellar structures and the SN compared to baseline. We found two significant clusters in the DMN (i.e., bilateral inferior parietal lobule) and one cluster in the LCEN (i.e., right inferior frontal gyrus). In these clusters, post-hoc comparisons in patients revealed a postictal decrease in within-network connectivity in the DMN and a postictal blunted decrease in LCEN within-network connectivity relative to healthy controls. However, these simple effects were non-significant.

**Table 3:** Voxel-wise results of resting-state networks of interest comparing changes over time in patients to those in healthy controls

	Network	Anatomical location based on Talairach and cerebellar atlas	Voxel cluster size	*p* value	MNI coordinates (x, y, z)		
Omnibus *F*-test	SN	Left Crus I	187	0.002	− 44	− 72	− 40
		Left anterior lobe V	178	0.015	− 4	− 58	− 24
		Right vermis	36	0.034	12	− 64	− 36
	DMN	Left inferior parietal lobule	15	0.040	44	− 52	58
		Right inferior parietal lobule	7	0.045	48	− 50	46
	LCEN	Right inferior frontal gyrus	307	0.006	52	24	− 6
Post-hoc comparisons							
*Patients* Postictal > baseline	SN	Right vermis V	23	0.026	4	− 58	− 24

*SN* salience network, *DMN* default mode network, *LCEN* left central executive network, *MNI* Montreal neurological institute

We did not establish baseline differences between patients and healthy controls nor a relation between connectivity changes and seizure duration, electrode placement, postictal midazolam or ROT in any of the networks. The other RSNs of interest, ATN and RCEN, did not show any significant changes in patients compared to those in healthy controls.

#### Other networks

No significant group by time interactions were observed in other RSNs.

## Discussion

This post hoc analysis of randomized clinical trial data investigated postictal changes in RSN functional connectivity in patients shortly after ECT-induced seizures, controlled for test–retest effects in healthy controls. We demonstrated decreases in postictal mean network connectivity strength in the LCEN and the auditory network. In addition, we found an increase in postictal voxel-wise connectivity between the SN and the cerebellum. Further, postictal within-network connectivity in the DMN and LCEN decreased. ECT thus appeared to reduce functional connectivity in multiple RSN during the postictal state. However, none of the clinical parameters, including ROT and postictal use of midazolam, were associated with these RSN changes.

Within our RSNs of interest, we found decreased mean connectivity changes in the (left) CEN, which may underlie postictal side-effects as disorientation, confusion, and memory disturbances. We did not, however, establish associations of these changing RSNs with the clinical outcome ROT nor the postictal use of midazolam (as proxy for postictal problems), possibly due to lack of power or because most patients were already reoriented at the time they received their postictal scan.

**Fig. 4:**
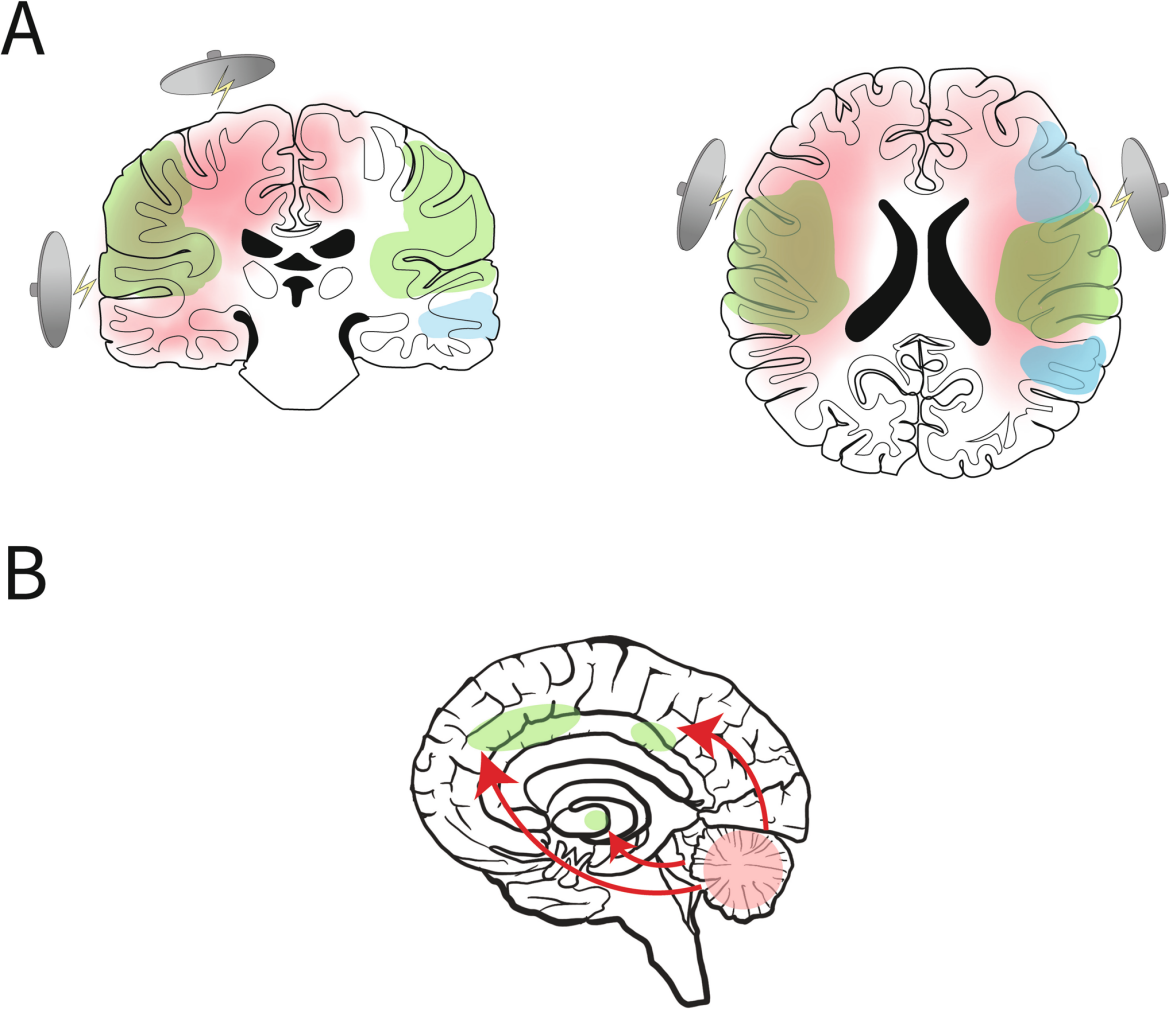
(**A**) Schematic representation of disruption of the left central executive network (blue) and the auditory network (green) induced by the electrical stimulus (red) administered with right unilateral (left) or bifrontotemporal (right) electrode placement, in electroconvulsive therapy. (**B**) Schematic depiction of increased postictal cerebellar (pink) connectivity with the salience network (green).

Additionally, we found decreased mean connectivity strength in the auditory network. One explanation for this result may be the position of the electrodes that deliver the electrical stimulus (i.e., UL or BL electrode placement). The auditory network comprises, amongst other, the right and left primary auditory cortex, lateral superior temporal gyrus and posterior insular cortex [33]. Possibly, these networks were disrupted due to the direct flow of electricity through these regions during the ECT-stimulus of a few seconds (see Fig. 4A) [34]. Involvement of the temporal lobe has been found to be crucial for development of cognitive side-effects in ECT [35].

In voxel-wise analyses, we established significant group by time interaction effects in the postictal state of the SN, DMN, and LCEN. In the SN analyses, we found increased postictal connectivity with cerebellar regions. This is interesting because the SN seems to be a critical connection between the DMN and CEN, ensuring proper balance between these networks [36]. It has been proposed that cerebellar circuits are involved in ECT-induced seizure termination by inhibiting generalized seizure activity in thalamocortical networks (Fig. 4B) [37]. It may be possible that the SN is increasingly recruited to serve as a postictal recovery process. Otherwise, in the DMN and LCEN, we showed decreased postictal within-network connectivity. Apparently, in the acute postictal state (i.e., one hour after seizures), the within-network DMN connectivity decreased. This postictal phenomenon may change over the course of a few weeks, because mixed findings have been reported after completed ECT-courses showing increases and decreases in between-network connectivity of the DMN and SN [9, 38]. Including more postictal rs-fMRI scans during the ECT-course in future studies may shed more light on these preliminary observations.

We did not find associations between RSNs connectivity changes and postictal clinical recovery (i.e., ROT and postictal use of midazolam), but our findings do match with our clinical observations in the postictal state. All our patients showed one or more postictal phenomena (i.e., fatigue, headache, disorientation, motor restlessness, altered consciousness, attention and memory problems), which leads to the assumption that network connectivity changes are related to the clinical presentation of the postictal state. It has been suggested that the CEN interacts with the DMN and the ATN to mediate memory and attention, which are both cognitive functions that are often impaired in the postictal state [39–41]. After the ECT-course, impaired attention, anterograde and retrograde amnesia are well-known cognitive side-effects, that may persist up to half a year after the treatment course [42, 43]. Dysfunction in the CEN may also be related to disorganization of thought, which may be associated with postictal psychosis [44–46].

We previously showed that postictal EEG recovery after ECT is comparable to that of patients with epilepsy, indicating that induced seizures are a useful model for epilepsy [18]. Functional connectivity after epileptic seizures, however, is challenging to investigate using MRI techniques, because of the unpredictability of spontaneous seizures. Given the similarities between ECT-induced seizures and (generalized) epileptic seizures, our MRI findings may also be informative for the postictal state in patients with epilepsy.

### Strengths and limitations

To our knowledge, this is the first study to systematically investigate RSNs in the immediate postictal state shortly after ECT-induced seizures. Strength of this research are the prospective protocolized collection of postictal MRI data and the ability to control for test–retest effects by including a large healthy control sample that was measured twice in the same MRI-scanner. Herewith, we show that systematic investigation of postictal RSNs in ECT patients is feasible. However, interpretation of our results is limited by the relatively small sample size. Furthermore, apart from the ECT stimulus or the seizure itself, decreased connectivity may have been influenced by the administered general anesthesia with etomidate. This is unlikely, however, as etomidate results in short anesthetic effects of several minutes after injection. It has been shown that propofol-induced loss of consciousness leads to decreased connectivity in the DMN and CEN [47]. However, at the time of rs-fMRI acquisition, most patients (76%, n = 13) were conscious and clinically reoriented (i.e., patients were aware of their personal information and current location), implying that anesthesia effects were presumably limited.

## Conclusion

ECT-induced seizures were associated with a postictal decrease of mean network connectivity strength in the left central executive and auditory networks and decreased within-network connectivity of the default mode and left central executive networks. There was increased postictal between-network connectivity between the salience network and cerebellum. These network changes may underlie clinical features of the postictal state.

## Supplementary Information

Below is the link to the electronic supplementary material.

Supplementary file1 (DOCX 963 KB)

## References

[1] Espinoza RT, Kellner CH (2022) Electroconvulsive therapy. N Engl J Med 386(7):667–672 35172057 10.1056/NEJMra2034954

[2] Medda P, Toni C, Perugi G (2014) The mood-stabilizing effects of electroconvulsive therapy. J ECT 30(4):275–282 25010031 10.1097/YCT.0000000000000160

[3] Datto CJ (2000) Side effects of electroconvulsive therapy. Depress Anxiety 12(3):130–134 11126187 10.1002/1520-6394(2000)12:3<130::AID-DA4>3.0.CO;2-C

[4] Palińska D (2007) SYMPOSIUM: ELECTROCONVULSIVE THERAPY. Consciousness impairment after electroconvulsive therapy. Curr Neurol 7(1):25

[5] Ingram A, Saling MM, Schweitzer I (2008) Cognitive side effects of brief pulse electroconvulsive therapy: a review. J ECT 24(1):3–9 18379328 10.1097/YCT.0b013e31815ef24a

[6] Fox MD, Raichle ME (2007) Spontaneous fluctuations in brain activity observed with functional magnetic resonance imaging. Nat Rev Neurosci 8(9):700–711 17704812 10.1038/nrn2201

[7] Rosazza C, Minati L (2011) Resting-state brain networks: literature review and clinical applications. Neurol Sci 32(5):773–785 21667095 10.1007/s10072-011-0636-y

[8] Jiang L, Geng W, Chen H, Zhang H, Bo F, Mao C-N, Chen Y-C, Yin X (2018) Decreased functional connectivity within the default-mode network in acute brainstem ischemic stroke. Eur J Radiol 105:221–226 30017284 10.1016/j.ejrad.2018.06.018

[9] Verdijk JP, van de Mortel LA, Ten Doesschate F, Pottkämper JC, Stuiver S, Bruin WB, Abbott CC, Argyelan M, Ousdal OT, Bartsch H (2024) Longitudinal resting-state network connectivity changes in electroconvulsive therapy patients compared to healthy controls. Brain Stimul 17(1):140–147 38101469 10.1016/j.brs.2023.12.005 PMC11145948

[10] Wang J, Wei Q, Wang L, Zhang H, Bai T, Cheng L, Tian Y, Wang K (2018) Functional reorganization of intra- and internetwork connectivity in major depressive disorder after electroconvulsive therapy. Hum Brain Mapp 39(3):1403–1411 29266749 10.1002/hbm.23928 PMC6866547

[11] Farrell JS, Gaxiola-Valdez I, Wolff MD, David LS, Dika HI, Geeraert BL, Rachel Wang X, Singh S, Spanswick SC, Dunn JF, Antle MC, Federico P, Teskey GC (2016) Postictal behavioural impairments are due to a severe prolonged hypoperfusion/hypoxia event that is COX-2 dependent. eLife 5:e19352 27874832 10.7554/eLife.19352 PMC5154758

[12] Pottkämper JC, Verdijk JP, Aalbregt E, Stuiver S, van de Mortel L, Norris DG, van Putten MJ, Hofmeijer J, Van Wingen GA, Van Waarde JA (2024) Changes in postictal cerebral perfusion are related to the duration of electroconvulsive therapy‐induced seizures. Epilepsia 65(1):177–189 10.1111/epi.17831 37973611

[13] DeSalvo MN, Schridde U, Mishra AM, Motelow JE, Purcaro MJ, Danielson N, Bai X, Hyder F, Blumenfeld H (2010) Focal BOLD fMRI changes in bicuculline-induced tonic–clonic seizures in the rat. Neuroimage 50(3):902–909 20079442 10.1016/j.neuroimage.2010.01.006 PMC2830359

[14] Verdijk JP, Pottkämper J, Verwijk E, van Wingen GA, van Putten MJ, Hofmeijer J, van Waarde JA (2022) Study of effect of nimodipine and acetaminophen on postictal symptoms in depressed patients after electroconvulsive therapy (SYNAPSE). Trials 23(1):1–15 35436940 10.1186/s13063-022-06206-y PMC9014277

[15] Lecrubier Y, Sheehan DV, Weiller E, Amorim P, Bonora I, Sheehan KH, Janavs J, Dunbar GC (1997) The Mini International Neuropsychiatric Interview (MINI). A short diagnostic structured interview: reliability and validity according to the CIDI. Eur Psychiatry 12(5):224–231

[16] Sobin C, Sackeim HA, Prudic J, Devanand DP, Moody BJ, McElhiney MC (1995) Predictors of retrograde amnesia following ECT. Am J Psychiatry 152:995–1001 7793470 10.1176/ajp.152.7.995

[17] d’Elia G (1970) Unilateral electroconvulsive therapy. Acta Psychiatr Scand 215:1–98 5271208

[18] Pottkämper JC, Verdijk JP, Hofmeijer J, van Waarde JA, van Putten MJ (2021) Seizures induced in electroconvulsive therapy as a human epilepsy model: a comparative case study. Epilepsia Open 6(4):672–684 34351710 10.1002/epi4.12532 PMC8633469

[19] Esteban O, Markiewicz CJ, Blair RW, Moodie CA, Isik AI, Erramuzpe A, Kent JD, Goncalves M, DuPre E, Snyder M (2019) fMRIPrep: a robust preprocessing pipeline for functional MRI. Nat Methods 16(1):111–116 30532080 10.1038/s41592-018-0235-4 PMC6319393

[20] Rachakonda S, Egolf E, Correa N, Calhoun V (2007) Group ICA of fMRI toolbox (GIFT) manual. Dostupnez [cit 2011-11-5]

[21] Du Y, Fan Y (2013) Group information guided ICA for fMRI data analysis. Neuroimage 69:157–197 23194820 10.1016/j.neuroimage.2012.11.008

[22] Du Y, Fu Z, Sui J, Gao S, Xing Y, Lin D, Salman M, Abrol A, Rahaman MA, Chen J (2020) NeuroMark: an automated and adaptive ICA based pipeline to identify reproducible fMRI markers of brain disorders. NeuroImage Clin 28:102375 32961402 10.1016/j.nicl.2020.102375 PMC7509081

[23] Iraji A, Deramus TP, Lewis N, Yaesoubi M, Stephen JM, Erhardt E, Belger A, Ford JM, McEwen S, Mathalon DH (2019) The spatial chronnectome reveals a dynamic interplay between functional segregation and integration. Hum Brain Mapp 40(10):3058–3077 30884018 10.1002/hbm.24580 PMC6548674

[24] Menon V (2011) Large-scale brain networks and psychopathology: a unifying triple network model. Trends Cogn Sci 15(10):483–506 21908230 10.1016/j.tics.2011.08.003

[25] Zheng H, Xu L, Xie F, Guo X, Zhang J, Yao L, Wu X (2015) The altered triple networks interaction in depression under resting state based on graph theory. BioMed Res Int 2015(1):386326 26180798 10.1155/2015/386326 PMC4477135

[26] Gao Y, Guo X, Zhong Y, Liu X, Tian S, Deng J, Lin X, Bao Y, Lu L, Wang G (2023) Decreased dorsal attention network homogeneity as a potential neuroimaging biomarker for major depressive disorder. J Affect Disord 332:136–142 36990286 10.1016/j.jad.2023.03.080

[27] Lv H, Wang Z, Tong E, Williams LM, Zaharchuk G, Zeineh M, Goldstein-Piekarski AN, Ball TM, Liao C, Wintermark M (2018) Resting-state functional MRI: everything that nonexperts have always wanted to know. Am J Neuroradiol 39(8):1390–1399 29348136 10.3174/ajnr.A5527 PMC6051935

[28] RStudio Team (2020) RStudio: integrated development for r. RStudio, PBC, Boston

[29] Bürkner P-C (2017) Advanced Bayesian multilevel modeling with the R package brms. arXiv preprint arXiv: 1705.11123

[30] Kruschke JK (2018) Rejecting or accepting parameter values in Bayesian estimation. Adv Methods Pract Psychol Sci 1(2):270–280

[31] Cohen J (1988) Statistical power anaylsis for the behavioral sciences

[32] Smith SM, Nichols TE (2009) Threshold-free cluster enhancement: addressing problems of smoothing, threshold dependence and localisation in cluster inference. Neuroimage 44(1):83–98 18501637 10.1016/j.neuroimage.2008.03.061

[33] Smitha K, Akhil Raja K, Arun K, Rajesh P, Thomas B, Kapilamoorthy T, Kesavadas C (2017) Resting state fMRI: A review on methods in resting state connectivity analysis and resting state networks. Neuroradiol J 30(4):305–317 28353416 10.1177/1971400917697342 PMC5524274

[34] Fridgeirsson EA, Deng Z-D, Denys D, van Waarde JA, van Wingen GA (2021) Electric field strength induced by electroconvulsive therapy is associated with clinical outcome. NeuroImage Clin 30:102581 33588322 10.1016/j.nicl.2021.102581 PMC7895836

[35] Blumenfeld H, Westerveld M, Ostroff RB, Vanderhill SD, Freeman J, Necochea A, Uranga P, Tanhehco T, Smith A, Seibyl JP (2003) Selective frontal, parietal, and temporal networks in generalized seizures. Neuroimage 19(4):1556–1566 12948711 10.1016/s1053-8119(03)00204-0

[36] Goulden N, Khusnulina A, Davis NJ, Bracewell RM, Bokde AL, McNulty JP, Mullins PG (2014) The salience network is responsible for switching between the default mode network and the central executive network: replication from DCM. Neuroimage 99:180–190 24862074 10.1016/j.neuroimage.2014.05.052

[37] Leaver AM, Espinoza R, Wade B, Narr KL (2022) Parsing the network mechanisms of electroconvulsive therapy. Biol Psychiatry 92(3):193–203 35120710 10.1016/j.biopsych.2021.11.016 PMC9196257

[38] Ten Doesschate F, Bruin W, Zeidman P, Abbott CC, Argyelan M, Dols A, Emsell L, van Eijndhoven PF, van Exel E, Mulders PC (2023) Effective resting-state connectivity in severe unipolar depression before and after electroconvulsive therapy. Brain Stimul 16(4):1128–1134 37517467 10.1016/j.brs.2023.07.054

[39] Wei H, An J, Zeng L, Shen H, Qiu S, Hu D (2015) Altered functional connectivity among default, attention, and control networks in idiopathic generalized epilepsy. Epilepsy Behav 46:118–125 25935514 10.1016/j.yebeh.2015.03.031

[40] Corbetta M, Patel G, Shulman GL (2008) The reorienting system of the human brain: From environment to theory of mind. Neuron 58(3):306–324 18466742 10.1016/j.neuron.2008.04.017 PMC2441869

[41] Cabeza R, Ciaramelli E, Olson IR, Moscovitch M (2008) The parietal cortex and episodic memory: an attentional account. Nat Rev Neurosci 9(8):613–625 18641668 10.1038/nrn2459 PMC2692883

[42] Sackeim HA (2000) Memory and ECT: from polarization to reconciliation. J ECT 16(2):87–96 10868319 10.1097/00124509-200006000-00001

[43] Verwijk E, Obbels J, Spaans H, Sienaert P (2017) Doctor, will I get my memory back? Electroconvulsive therapy and cognitive side-effects in daily practice. Tijdschr Psychiatr 59:632–637 29077139

[44] Cataldi M, Avoli M, de Villers-Sidani E (2013) Resting state networks in temporal lobe epilepsy. Epilepsia 54(12):2048–2059 24117098 10.1111/epi.12400 PMC4880458

[45] Canuet L, Ishii R, Iwase M, Ikezawa K, Kurimoto R, Takahashi H, Currais A, Azechi M, Aoki Y, Nakahachi T (2011) Psychopathology and working memory-induced activation of the prefrontal cortex in schizophrenia-like psychosis of epilepsy: evidence from magnetoencephalography. Psychiatry Clin Neurosci 65(2):183–190 21414092 10.1111/j.1440-1819.2010.02179.x

[46] Canuet L, Ishii R, Pascual-Marqui RD, Iwase M, Kurimoto R, Aoki Y, Ikeda S, Takahashi H, Nakahachi T, Takeda M (2011) Resting-state EEG source localization and functional connectivity in schizophrenia-like psychosis of epilepsy. PLoS ONE 6(11):e27863 22125634 10.1371/journal.pone.0027863 PMC3220705

[47] Boveroux P, Vanhaudenhuyse A, Bruno M-A, Noirhomme Q, Lauwick S, Luxen A, Degueldre C, Plenevaux A, Schnakers C, Phillips C (2010) Breakdown of within-and between-network resting state functional magnetic resonance imaging connectivity during propofol-induced loss of consciousness. J Am Soc Anesthesiol 113(5):1038–1053 10.1097/ALN.0b013e3181f697f5 20885292

