# Supplementary material for: Postictal resting-state connectivity changes after electroconvulsive therapy-induced seizures
Source: Eur Arch Psychiatry Clin Neurosci. 2025 Jul 14;276(2):851–62. doi: 10.1007/s00406-025-02043-7 (PMC12953249; doi:10.1007/s00406-025-02043-7)
Supplement: Supplementary file 1 — Supplementary file1 (DOCX 963 KB) [file 406_2025_2043_MOESM1_ESM.docx]

**Supplementary materials**

**Methods**

**Electroencephalography**

Ictal electroencephalography (EEG) was used to determined seizure duration. Twelve silver/silver chloride cup electrodes were applied according to the international 10-20 system. EEGs were recorded using a NeuroCenter EEG recording system (Clinical Science Systems) with a full-band DC-coupled amplifier (TMSi). Seizure duration was determined visually and defined as the time interval in seconds between the onset of rhythmicity or spike-wave complexes and the onset of postictal generalized suppression in all channels. More details are provided elsewhere.[1, 2]

**Preprocessing**

Results included in this manuscript come from preprocessing performed using *fMRIPrep* 21.0.2, which is based on *Nipype* 1.6.1. [3, 4-6]

**Preprocessing of B_0_ inhomogeneity mappings**

*B_0_*-nonuniformity map (or fieldmap) were estimated based on two (or more) echo-planar imaging (EPI) references with *topup*.[7]

**Anatomical data preprocessing**

T1-weighted (T1w) images were corrected for intensity non-uniformity (INU) with M4BiasFieldCorrection, distributed with ANTs 2.3.3, and used as T1w-reference throughout the workflow.[8, 9] The T1w-reference was then skull-stripped with a *Nipype* implementation of the antsBrainExtration.sh workflow (from ANTs), using OASIS30ANTs as target template. Brain tissue segmentation of cerebrospinal fluid (CSF), white-matter (WM), and gray-matter (GM) was performed on the brain-extracted T1w using *fast* (FSL 6.0.5.1).[10] Volume-based spatial normalization to two standard spaces (MNI152NLin2009cAsym, MNI152NLin6Asym) was performed through nonlinear registration with *antsRegistration* (ANTs 2.3.3), using brain-extracted versions of both T1w reference and the T1w template. The following templates were selected for spatial normalization: *ICBM 152 Nonlinear Asymmetrical template version 2009c* [TemplateFlow ID: MNI152NLin2009cAsym] and *FSL’s MNI ICBM 152 non-linear 6th Generation Asymmetric Average Brain Stereotaxic Registration Model* [TemplateFlow ID: MNI152NLin6Asym].[11, 12]

**Functional data preprocessing**

For each of the BOLD runs per subject, the following preprocessing was performed. First, a reference volume and its skull-stripped version were generated using a custom methodology of *fMRIPrep*. Head-motion parameters with respect to the BOLD reference (transformation matrices, and six corresponding rotation and translation parameters) are estimated before any spatiotemporal filtering using *mcflirt*.[13] The estimated fieldmap was then aligned with rigid-registration to the target EPI (echo-planar imaging) reference run. The field coefficients were mapped on to the reference EPI using the transform. The BOLD reference was then co-registered to the T1w reference using *mri_coreg* (FreeSurfer) followed by *flirt* with the boundary-based registration cost-function.[14, 15] Co-registration was configured with six degrees of freedom. Several confounding time-series were calculated based on the *preprocessed BOLD*: framewise displacement (FD), DVARS, and three region-wise global signals. FD was computed using two formulations following Power et al. (absolute sum of relative motions) and Jenkinson et al. (relative root mean square displacement between affines).[13, 16] FD and DVARS are calculated for each functional run, both using their implementations in *Nipype* (following the definitions by Power et al.).[16] The three global signals are extracted within the CSF, the WM, and the whole-brain masks. Additionally, a set of physiological regressors were extracted to allow for component-based noise correction (*CompCor*).[17] Principal components are estimated after high-pass filtering the *preprocessed BOLD* time-series (using a discrete cosine filter with 128s cut-off) for the two *CompCor* variants: temporal (tCompCor) and anatomical (aCompCor). tCompCor components are then calculated from the top 2% variable voxels within the brain mask. For aCompCor, three probabilistic masks (CSF, WM and combined CSF+WM) are generated in anatomical space. The implementation differs from that of Behzadi et al. in that instead of eroding the masks by 2 pixels on BOLD space, the aCompCor masks are subtracted a mask of pixels that likely contain a volume fraction of GM. This mask is obtained by thresholding the corresponding partial volume map at 0.05, and it ensures components are not extracted from voxels containing a minimal fraction of GM. Finally, these masks are resampled into BOLD space and binarized by thresholding at 0.99 (as in the original implementation). Components are also calculated separately within the WM and CSF masks. For each CompCor decomposition, the *k* components with the largest singular values are retained, such that the retained components’ time series are sufficient to explain 50 percent of variance across the nuisance mask (CSF, WM, combined, or temporal). The remaining components are dropped from consideration. The head-motion estimates calculated in the correction step were also placed within the corresponding confounds file. The confound time series derived from head motion estimates and global signals were expanded with the inclusion of temporal derivatives and quadratic terms for each.[18] Frames that exceeded a threshold of 0.5 mm FD or 1.5 standardized DVARS were annotated as motion outliers. The BOLD time-series were resampled into standard space, generating a preprocessed BOLD run in MNI152NLin2009cAsym space. First, a reference volume and its skull-stripped version were generated using a custom methodology of *fMRIPrep*. Automatic removal of motion artifacts using independent component analysis (ICA-AROMA) was performed on the *preprocessed BOLD on MNI space* time-series after removal of non-steady state volumes and spatial smoothing with an isotropic, Gaussian kernel of 6mm FWHM (full-width half-maximum).[19] Corresponding “non-aggressively” denoised runs were produced after such smoothing. Additionally, the “aggressive” noise-regressors were collected and placed in the corresponding confounds file. All resamplings can be performed with a single interpolation step by composing all the pertinent transformations (i.e., head-motion transform matrices, susceptibility distortion correction when available, and co-registrations to anatomical and output spaces). Gridded (volumetric) resamplings were performed using *antsApplyTransforms* (ANTs), configured with Lanczos interpolation to minimize the smoothing effects of other kernels.[20] Non-gridded (surface) resamplings were performed using *mri_vol2surf* (FreeSurfer).

Many internal operations of *fMRIPrep* use *Nilearn* 0.8.1, mostly within the functional processing workflow.[21] For more details of the pipeline, see (https://fmriprep.org/en/latest/workflows.html).

**Supplementary Table S1.** Bayesian mixed model results in patients showing decreased postictal mean network connectivity strength in the left central executive networks and auditory network

|  | **ATN** | | **DMN** | | **LCEN** | | **RCEN** | | **SN** | | **AUD** | |
| --- | --- | --- | --- | --- | --- | --- | --- | --- | --- | --- | --- | --- |
| *Predictors* | *Estimates* | *CrI (95%)* | *Estimates* | *CrI (95%)* | *Estimates* | *CrI (95%)* | *Estimates* | *CrI (95%)* | *Estimates* | *CrI (95%)* | *Estimates* | *CrI (95%)* |
| Intercept | 1.17 | 0.03 – 2.33 | 2.08 | 1.23 – 2.90 | 1.93 | 0.98 – 2.86 | 2.18 | 1.51 – 2.85 | 0.24 | -0.31 – 0.78 | 2.17 | 1.23 – 3.18 |
| Session (Postictal) | -0.04 | -0.17 – 0.09 | -0.09 | -0.21 – 0.05 | -0.15 | -0.25 – -0.05* | -0.08 | -0.19 – 0.03 | -0.03 | -0.12 – 0.05 | -0.16 | -0.29 – -0.04* |
| Age (years) | -0.00 | -0.01 – 0.01 | -0.00 | -0.01 – 0.00 | -0.00 | -0.01 – 0.01 | -0.00 | -0.01 – 0.00 | -0.00 | -0.01 – 0.00 | -0.00 | -0.01 – 0.00 |
| Sex (Female) | -0.09 | -0.30 – 0.13 | -0.05 | -0.21 – 0.11 | -0.06 | -0.25 – 0.13 | -0.18 | -0.32 – -0.05* | -0.07 | -0.18 – 0.04 | -0.15 | -0.36 – 0.03 |
| Seizure duration (s) | 0.00 | -0.00 – 0.01 | 0.00 | -0.00 – 0.01 | 0.00 | -0.00 – 0.01 | 0.00 | -0.00 – 0.00 | 0.00 | -0.00 – 0.00 | 0.00 | -0.00 – 0.01 |
| Electrode placement (UL) | 0.07 | -0.15 – 0.30 | 0.11 | -0.05 – 0.28 | 0.04 | -0.13 – 0.23 | 0.08 | -0.06 – 0.22 | -0.03 | -0.14 – 0.09 | 0.19 | -0.00 – 0.39 |
| Δt ECT-stimulus – rs-fMRI (min) | 0.01 | -0.01 – 0.02 | 0.00 | -0.01 – 0.01 | 0.00 | -0.01 – 0.01 | 0.00 | -0.01 – 0.01 | 0.00 | -0.00 – 0.01 | 0.00 | -0.01 – 0.01 |
| ROT (min) | 0.00 | -0.00 – 0.01 | 0.00 | -0.01 – 0.01 | 0.00 | -0.01 – 0.01 | 0.00 | -0.01 – 0.01 | 0.00 | -0.01 – 0.00 | 0.00 | -0.01 – 0.00 |
| R^2^ |  |  |  |  | 0.34 | 0.15 – 0.50 | 0.41 | 0.22 – 0.55 |  |  | 0.44 | 0.25 – 0.58 |
| *ROPE  Interpretation |  | |  | | Credible | | Credible | |  | | Credible | |

ATN = attention network, AUD = auditory network, DMN = default mode network, LFPN = left central executive network, RCEN = right central executive network, SN = salience network, ROPE = region of practical equivalence, CI = credibility interval, UL = unilateral, Δt ECT-stimulus – rs-fMRI = time interval between the electroconvulsive therapy stimulus and acquisition of resting state functional magnetic resonance imaging, ROT = reorientation time.

**Supplementary Table S2.** Bayesian mixed model results of the influence of postictal midazolam administration on changes of mean network connectivity strength in any of the RSNs

|  | **ATN** | | **DMN** | | **LCEN** | | **RCEN** | | **SN** | | **AUD** | |
| --- | --- | --- | --- | --- | --- | --- | --- | --- | --- | --- | --- | --- |
| *Predictors* | *Estimates* | *CrI (95%)* | *Estimates* | *CrI (95%)* | *Estimates* | *CrI (95%)* | *Estimates* | *CrI (95%)* | *Estimates* | *CrI (95%)* | *Estimates* | *CrI (95%)* |
| Intercept | 0.00 | -0.89 – 0.94 | -0.05 | -1.11 – 0.93 | -0.25 | -0.96 – 0.45 | -0.22 | -1.15 – 0.65 | 0.02 | -0.60 – 0.63 | -0.16 | -1.15 – 0.78 |
| Age (years) | 0.00 | -0.01 – 0.01 | -0.00 | -0.01 – 0.01 | 0.00 | -0.01 – 0.01 | 0.00 | -0.01 – 0.01 | -0.00 | -0.01 – 0.00 | 0.00 | -0.01 – 0.01 |
| Sex (Female) | -0.01 | -0.44 – 0.40 | -0.05 | -0.51 – 0.41 | -0.08 | -0.40 – 0.24 | 0.02 | -0.40 – 0.42 | 0.16 | -0.12 – 0.45 | -0.02 | -0.46 – 0.42 |
| Postictal midazolam (Yes) | -0.11 | -0.54 – 0.31 | 0.01 | -0.45 – 0.48 | 0.00 | -0.32 – 0.35 | -0.00 | -0.45 – 0.41 | 0.13 | -0.14 – 0.43 | -0.06 | -0.53 – 0.38 |

ATN = attention network, AUD = auditory network, DMN = default mode network, LFPN = left central executive network, RCEN = right central executive network, SN = salience network, ROPE = region of practical equivalence, CI = credibility interval.

|  | **Cereb** | | **LANG** | | **MTR** | | **Sub** | | **VisPri** | | **VisSec** | |
| --- | --- | --- | --- | --- | --- | --- | --- | --- | --- | --- | --- | --- |
| *Predictors* | *Estimates* | *CrI (95%)* | *Estimates* | *CrI (95%)* | *Estimates* | *CrI (95%)* | *Estimates* | *CrI (95%)* | *Estimates* | *CrI (95%)* | *Estimates* | *CrI (95%)* |
| Intercept | -0.69 | -2.26 – 0.86 | 0.21 | -0.69 – 1.17 | 0.18 | -0.89 – 1.24 | 0.02 | -0.72 – 0.78 | 0.15 | -0.97 – 1.24 | 0.40 | -0.79 – 1.64 |
| Age (years) | 0.01 | -0.01 – 0.01 | -0.00 | -0.01 – 0.01 | -0.00 | -0.01 – 0.01 | -0.00 | -0.01 – 0.01 | -0.00 | -0.02 – 0.01 | -0.00 | -0.02 – 0.01 |
| Sex (Female) | 0.28 | -0.47 – 1.05 | -0.13 | -0.58 – 0.29 | -0.17 | -0.68 – 0.32 | 0.11 | -0.23 – 0.47 | -0.03 | -0.53 – 0.46 | 0.00 | -0.57 – 0.55 |
| Postictal midazolam (Yes) | 0.01 | -0.75 – 0.80 | -0.08 | -0.53 – 0.34 | -0.07 | -0.57 – 0.44 | 0.04 | -0.31 – 0.41 | 0.01 | -0.52 – 0.53 | -0.00 | -0.56 – 0.57 |

Cereb = Cerebellar network, LANG = language network, MTR = somatosensory network, Sub = subcortical network, VisPri = primary visual network, VisSec = secondary visual network, ROPE = region of practical equivalence, CI = credibility interval.

**Supplementary Table S3.** Bayesian regression results of patients and healthy controls showing no group by time interaction effects in any of the remaining RSNs

|  | **Cereb** | | **LANG** | | **MTR** | | **Sub** | | **VisPri** | | **VisSec** | |
| --- | --- | --- | --- | --- | --- | --- | --- | --- | --- | --- | --- | --- |
| *Predictors* | *Estimates* | *CrI (95%)* | *Estimates* | *CrI (95%)* | *Estimates* | *CrI (95%)* | *Estimates* | *CrI (95%)* | *Estimates* | *CrI (95%)* | *Estimates* | *CrI (95%)* |
| Intercept | -0.60 | -1.07 – -0.14 | -0.07 | -0.34 – 0.22 | -0.05 | -0.35 – 0.25 | 0.05 | -0.18 – 0.26 | 0.04 | -0.37 – 0.45 | 0.01 | -0.47 – 0.48 |
| Group (Patients) | -0.00 | -0.23 – 0.23 | -0.09 | -0.23 – 0.05 | -0.01 | -0.15 – 0.14 | 0.02 | -0.09 – 0.12 | -0.04 | -0.23 – 0.16 | 0.14 | -0.10 – 0.38 |
| Age (years) | 0.01 | 0.00 – 0.02 | 0.00 | -0.00 – 0.01 | 0.00 | -0.00 – 0.01 | -0.00 | -0.00 – 0.00 | -0.00 | -0.01 – 0.01 | -0.00 | -0.01 – 0.01 |
| Sex (Female) | 0.16 | -0.07 – 0.38 | 0.01 | -0.14 – 0.15 | -0.03 | -0.17 – 0.11 | 0.03 | -0.08 – 0.13 | 0.05 | -0.14 – 0.24 | 0.08 | -0.16 – 0.32 |

Cereb = Cerebellar network, LANG = language network, MTR = somatosensory network, Sub = subcortical network, VisPri = primary visual network, VisSec = secondary visual network, ROPE = region of practical equivalence, CI = credibility interval.

**Supplementary Table S4.** Bayesian mixed model results in patients showing decreased postictal mean network connectivity strength in females in language network

|  | **Cereb** | | | | | | **LANG** | | **MTR** | | **Sub** | | **VisPri** | | **VisSec** | |
| --- | --- | --- | --- | --- | --- | --- | --- | --- | --- | --- | --- | --- | --- | --- | --- | --- |
| *Predictors* | *Estimates* | | *CrI (95%)* | | | | *Estimates* | *CrI (95%)* | *Estimates* | *CrI (95%)* | *Estimates* | *CrI (95%)* | *Estimates* | *CrI (95%)* | *Estimates* | *CrI (95%)* |
| Intercept | 1.60 | | 0.43 – 2.85 | | | | 2.41 | 1.65 – 3.21 | 2.05 | 1.03 – 3.05 | 0.30 | -0.50 – 1.13 | 3.38 | 2.27 – 4.54 | 2.72 | 1.47 – 3.96 |
| Session (Postictal) | 0.02 | | -0.18 – 0.22 | | | | -0.08 | -0.20 – 0.04 | 0.00 | -0.14 – 0.15 | 0.02 | -0.08 – 0.12 | -0.01 | -0.16 – 0.14 | 0.14 | -0.03 – 0.31 |
| Age (years) | -0.00 | | -0.01 – 0.01 | | | | -0.00 | -0.01 – 0.00 | -0.00 | -0.01 – 0.01 | -0.00 | -0.01 – 0.00 | -0.01 | -0.02 – 0.00 | -0.01 | -0.02 – 0.00 |
| Sex (Female) | -0.24 | | -0.48 – -0.01* | | | | -0.24 | -0.39 – -0.08* | -0.06 | -0.25 – 0.14 | -0.12 | -0.28 – 0.04 | -0.16 | -0.40 – 0.10 | -0.21 | -0.45 – 0.04 |
| Seizure duration (s) | 0.00 | | -0.00 – 0.01 | | | | 0.00 | -0.00 – 0.00 | 0.00 | -0.00 – 0.01 | -0.00 | -0.00 – 0.00 | -0.00 | -0.01 – 0.00 | -0.00 | -0.01 – 0.01 |
| Electrode placement (UL) | 0.04 | | -0.20 – 0.28 | | | | -0.03 | -0.19 – 0.13 | -0.04 | -0.25 – 0.15 | -0.07 | -0.24 – 0.09 | -0.05 | -0.28 – 0.19 | -0.10 | -0.35 – 0.16 |
| Δt ECT-stimulus – rs-fMRI (min) | 0.01 | | -0.01 – 0.02 | | | | -0.00 | -0.01 – 0.01 | 0.00 | -0.01 – 0.01 | 0.00 | -0.01 – 0.01 | -0.00 | -0.01 – 0.01 | 0.00 | -0.01 – 0.02 |
| ROT (min) | 0.00 | | -0.01 – 0.00 | | | | -0.00 | -0.01 – 0.01 | -0.00 | -0.01 – 0.00 | 0.00 | -0.01 – 0.00 | 0.00 | -0.01 – 0.00 | -0.00 | -0.01 – 0.01 |
| *R^2^ | 0.33 | | | 0.15 – 0.50 | | | 0.37 | 0.19 – 0.52 |  | |  | |  | |  | |
| *ROPE interpretation | Undecided | | | | | | Credible | |  | |  | |  | |  | |
|  |  |  |  | |  |  |  |  |  |  |  |  |  |  |  |  |

Cereb = Cerebellar network, LANG = language network, MTR = somatosensory network, Sub = subcortical network, VisPri = primary visual network, VisSec = secondary visual network, ROPE = region of practical equivalence, CI = credibility interval, UL = unilateral, Δt ECT-stimulus – rs-fMRI = time interval between the electroconvulsive therapy stimulus and acquisition of resting state functional magnetic resonance imaging, ROT = reorientation time.

**Supplementary Table S5.** Bayesian mixed model results of healthy controls showing no changes of mean network connectivity strength in any of the RSNs

|  | **ATN** | | **AUD** | | **DMN** | | **LCEN** | | **RCEN** | | **SN** | |
| --- | --- | --- | --- | --- | --- | --- | --- | --- | --- | --- | --- | --- |
| *Predictors* | *Estimates* | *CrI (95%)* | *Estimates* | *CrI (95%)* | *Estimates* | *CrI (95%)* | *Estimates* | *CrI (95%)* | *Estimates* | *CrI (95%)* | *Estimates* | *CrI (95%)* |
| Intercept | 1.84 | 1.65 – 2.02 | 2.17 | 1.95 – 2.38 | 2.31 | 2.15 – 2.46 | 2.13 | 1.98 – 2.29 | 2.20 | 2.03 – 2.38 | 0.27 | 0.17 – 0.38 |
| Session (Follow-up) | 0.02 | -0.07 – 0.12 | 0.04 | -0.07 – 0.14 | -0.03 | -0.11 – 0.05 | 0.02 | -0.05 – 0.10 | 0.04 | -0.05 – 0.13 | -0.02 | -0.07 – 0.04 |
| Age (years) | -0.00 | -0.01 – 0.01 | -0.00 | -0.00 – 0.01 | -0.00 | -0.00 – 0.00 | -0.00 | -0.01 – 0.00 | -0.00 | -0.00 – 0.00 | 0.00 | -0.00 – 0.00 |
| Sex (Female) | -0.06 | -0.16 – 0.04 | -0.05 | -0.15 – 0.06 | 0.05 | -0.03 – 0.12 | 0.01 | -0.07 – 0.09 | -0.08 | -0.18 – 0.01 | -0.03 | -0.08 – 0.03 |

ATN = attention network, AUD = auditory network, DMN = default mode network, LFPN = left central executive network, RCEN = right central executive network, SN = salience network, ROPE = region of practical equivalence, CI = credibility interval.

|  | **Cereb** | | **LANG** | | **MTR** | | **Sub** | | **VisPri** | | **VisSec** | |
| --- | --- | --- | --- | --- | --- | --- | --- | --- | --- | --- | --- | --- |
| *Predictors* | *Estimates* | *CrI (95%)* | *Estimates* | *CrI (95%)* | *Estimates* | *CrI (95%)* | *Estimates* | *CrI (95%)* | *Estimates* | *CrI (95%)* | *Estimates* | *CrI (95%)* |
| Intercept | 2.30 | 1.97 – 2.61 | 1.82 | 1.61 – 2.03 | 1.96 | 1.78 – 2.13 | 0.23 | 0.08 – 0.37 | 2.68 | 2.36 – 2.99 | 2.17 | 1.82 – 2.54 |
| Session (Follow-up) | -0.03 | -0.19 – 0.13 | 0.00 | -0.10 – 0.11 | -0.01 | -0.08 – 0.09 | 0.01 | -0.06 – 0.08 | 0.03 | -0.13 – 0.19 | -0.01 | -0.19 – 0.18 |
| Age (years) | -0.00 | -0.01 – 0.01 | 0.00 | -0.00 – 0.00 | -0.00 | -0.00 – 0.00 | -0.00 | -0.00 – 0.00 | -0.00 | -0.00 – 0.01 | 0.00 | -0.00 – 0.01 |
| Sex (Female) | -0.25 | -0.41 – -0.07* | -0.01 | -0.12 – 0.09 | -0.10 | -0.18 – -0.01* | 0.05 | -0.03 – 0.12 | -0.15 | -0.31 – 0.01 | -0.20 | -0.38 – -0.01* |
| *R^2^ | 0.18 | 0.04 – 0.33 |  |  | 0.12 | 0.01 – 0.25 |  |  |  |  | 0.14 | 0.01 – 0.28 |
| *ROPE interpretation | Rejected | |  |  | Undecided | |  |  |  |  | Undecided | |

Cereb = Cerebellar network, LANG = language network, MTR = somatosensory network, Sub = subcortical network, VisPri = primary visual network, VisSec = secondary visual network, ROPE = region of practical equivalence, CI = credibility interval.

|  |  |  |  |  |  |  |
| --- | --- | --- | --- | --- | --- | --- |


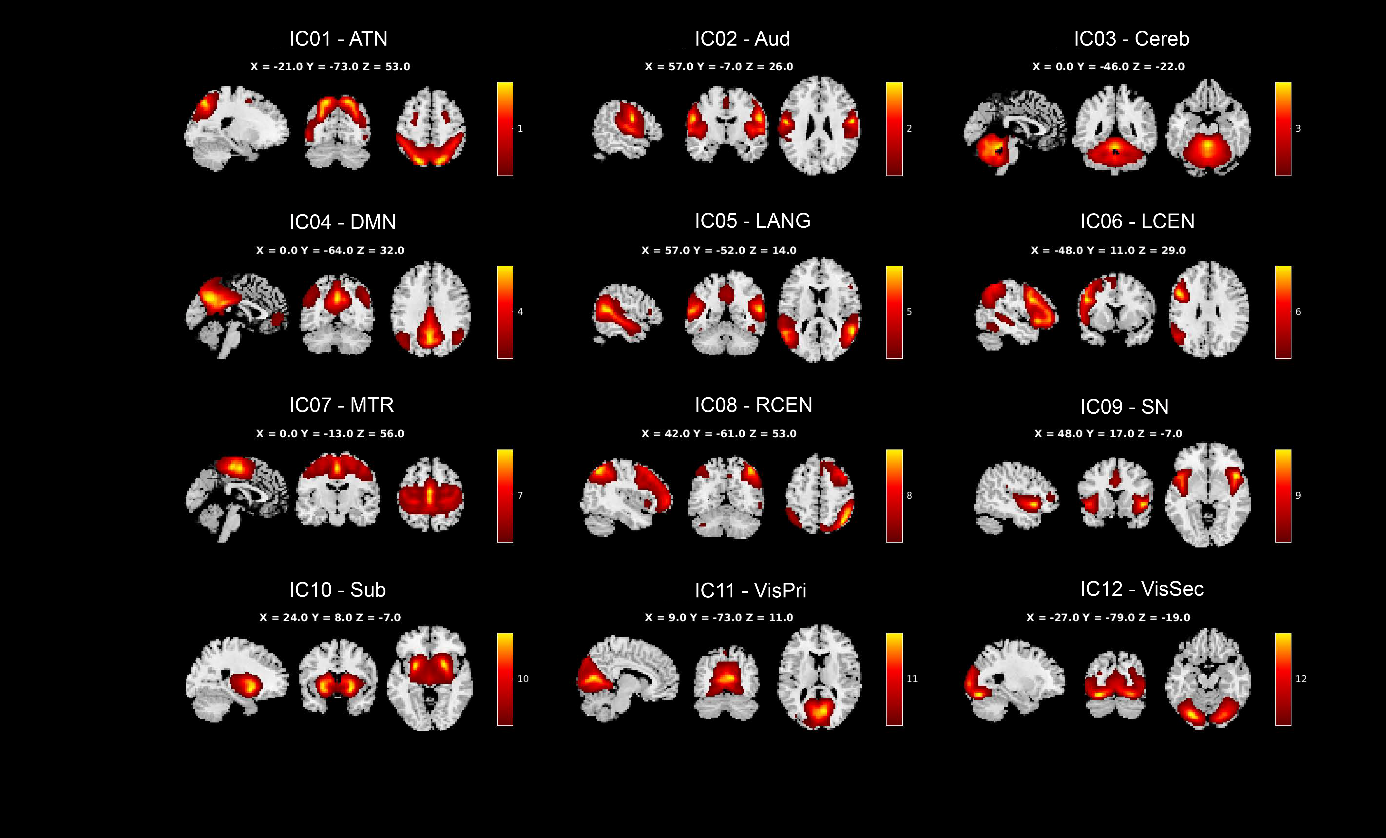


**Figure S1.** Overview of all RSN templates based on an independent sample of healthy controls (n = 160). ATN = attention network, Aud = auditory network, Cereb = Cerebellar network, DMN = default mode network, LANG = language network, LCEN = left central executive network, MTR = somatosensory network, RCEN = right central executive network, SN = salience network, Sub = subcortical network, VisPri = primary visual network, VisSec = secondary visual network


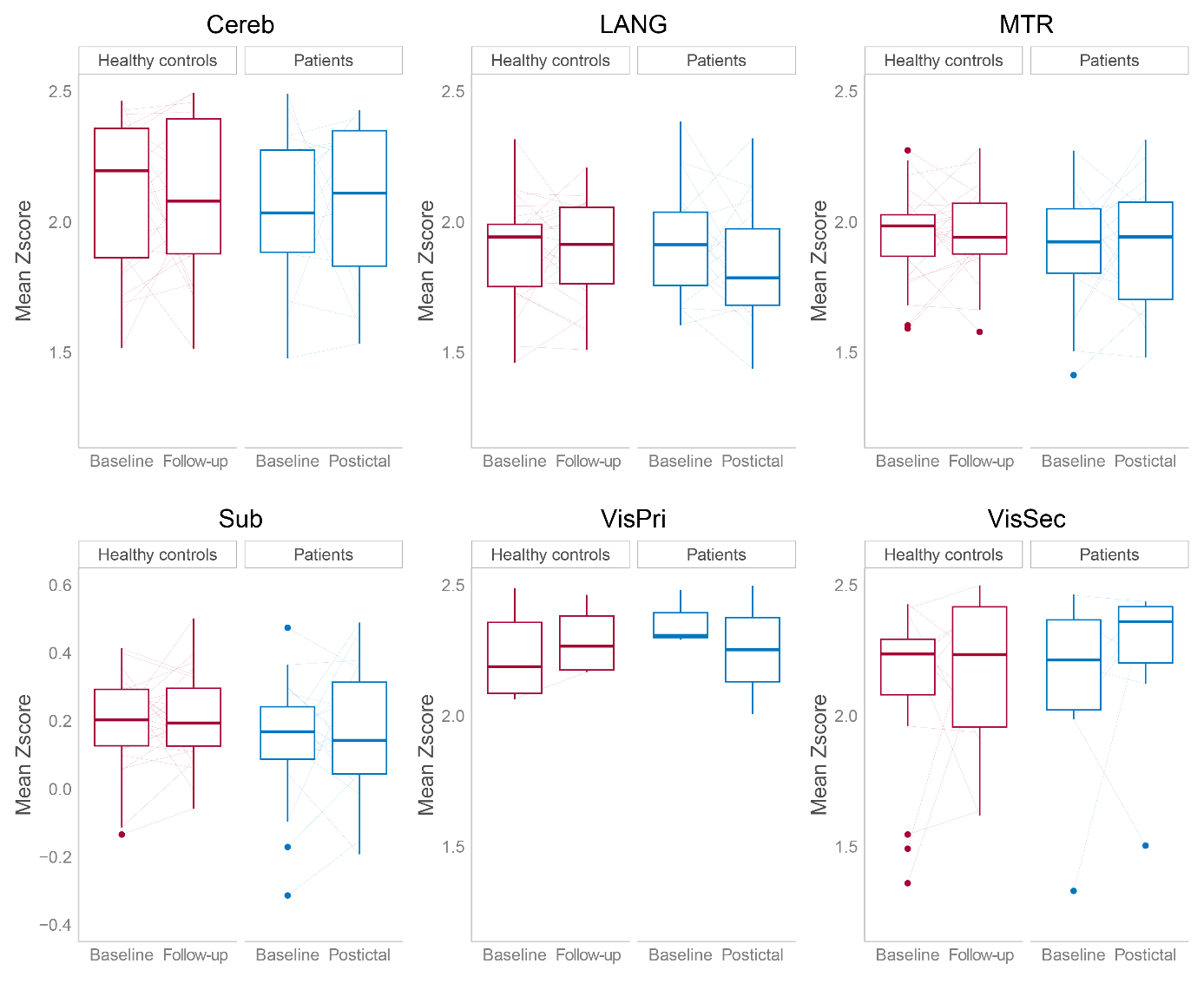


**Figure S2**. Postictal mean network connectivity in exploratory resting-state networks (RSNs) that did not show any changes compared to baseline in electroconvulsive therapy patients (n = 17) compared to healthy controls (n = 27). Cereb = cerebellar network, LANG = language network, MTR = sensorimotor network, Sub = subcortical network, VisPri = primary visual network, VisSec = secondary visual network.

**References**

1. Pottkämper JC, Verdijk JP, Hofmeijer J, van Waarde JA, van Putten MJ. Seizures induced in electroconvulsive therapy as a human epilepsy model: A comparative case study. Epilepsia Open. 2021;6(4):672-84.

2. Esteban O, Markiewicz CJ, Blair RW, Moodie CA, Isik AI, Erramuzpe A, et al. fMRIPrep: a robust preprocessing pipeline for functional MRI. Nat Methods. 2019;16(1):111-6.

3. Pottkämper JC, Verdijk JP, Aalbregt E, Stuiver S, van de Morte L, Norris DG, et al. Changes of postictal cerebral perfusion are related to the duration of electroconvulsive therapy-induced seizures. 2023.

4. Esteban O, Blair R, Markiewicz C, Berleant S, Moodie C, Ma F, et al. fMRIPrep. Software. Zenodo. 2018.

5. Gorgolewski KJ, Esteban O, Markiewicz CJ, Ziegler E, Ellis DG, Notter MP, et al. Nipype. Software. 2018.

6. Gorgolewski K, Burns CD, Madison C, Clark D, Halchenko YO, Waskom ML, et al. Nipype: a flexible, lightweight and extensible neuroimaging data processing framework in python. Front Neuroinform. 2011:13.

7. Andersson JL, Skare S, Ashburner J. How to correct susceptibility distortions in spin-echo echo-planar images: application to diffusion tensor imaging. Neuroimage. 2003;20(2):870-88.

8. Tustison NJ, Avants BB, Cook PA, Zheng Y, Egan A, Yushkevich PA, et al. N4ITK: improved N3 bias correction. IEEE Trans Med Imaging. 2010;29(6):1310-20.

9. Avants BB, Epstein CL, Grossman M, Gee JC. Symmetric diffeomorphic image registration with cross-correlation: evaluating automated labeling of elderly and neurodegenerative brain. Med Image Anal. 2008;12(1):26-41.

10. Zhang Y, Brady M, Smith S. Segmentation of brain MR images through a hidden Markov random field model and the expectation-maximization algorithm. IEEE Trans Med Imaging. 2001;20(1):45-57.

11. Evans AC, Janke AL, Collins DL, Baillet S. Brain templates and atlases. Neuroimage. 2012;62(2):911-22.

12. Fonov VS, Evans AC, McKinstry RC, Almli CR, Collins D. Unbiased nonlinear average age-appropriate brain templates from birth to adulthood. Neuroimage. 2009(47):S102.

13. Jenkinson M, Bannister P, Brady M, Smith S. Improved optimization for the robust and accurate linear registration and motion correction of brain images. Neuroimage. 2002;17(2):825-41.

14. Jenkinson M, Smith S. A global optimisation method for robust affine registration of brain images. Med Image Anal. 2001;5(2):143-56.

15. Greve DN, Fischl B. Accurate and robust brain image alignment using boundary-based registration. Neuroimage. 2009;48(1):63-72.

16. Power JD, Mitra A, Laumann TO, Snyder AZ, Schlaggar BL, Petersen SE. Methods to detect, characterize, and remove motion artifact in resting state fMRI. Neuroimage. 2014;84:320-41.

17. Behzadi Y, Restom K, Liau J, Liu TT. A component based noise correction method (CompCor) for BOLD and perfusion based fMRI. Neuroimage. 2007;37(1):90-101.

18. Satterthwaite TD, Elliott MA, Gerraty RT, Ruparel K, Loughead J, Calkins ME, et al. An improved framework for confound regression and filtering for control of motion artifact in the preprocessing of resting-state functional connectivity data. Neuroimage. 2013;64:240-56.

19. Pruim RH, Mennes M, van Rooij D, Llera A, Buitelaar JK, Beckmann CF. ICA-AROMA: A robust ICA-based strategy for removing motion artifacts from fMRI data. Neuroimage. 2015;112:267-77.

20. Lanczos C. Evaluation of noisy data. Journal of the Society for Industrial and Applied Mathematics, Series B: Numerical Analysis. 1964;1(1):76-85.

21. Abraham A, Pedregosa F, Eickenberg M, Gervais P, Mueller A, Kossaifi J, et al. Machine learning for neuroimaging with scikit-learn. Front Neuroinform. 2014:14.
